# Optimizing drying techniques for turmeric (*Curcuma longa* L.): impacts on color, curcumin, and essential oil composition

**DOI:** 10.1016/j.fochx.2025.102914

**Published:** 2025-08-20

**Authors:** Aghdas Shahimoridi, Mohammad-Taghi Ebadi, Mahdi Ayyari, Yadollah Yamini

**Affiliations:** a Department of Horticultural Science, Faculty of Agriculture, Tarbiat Modares University, Tehran, Iran; b Department of Chemistry, Faculty of Basic Sciences, Tarbiat Modares University, Tehran, Iran

**Keywords:** Turmeric, Drying, Curcumin, Color, Essential oil

## Abstract

Turmeric (*Curcuma longa L.*) is primarily traded in dried form; preserving its bioactive compounds and quality through optimal drying methods is critical. This study evaluated the effects of various drying techniques—sun drying (SD), convection oven drying (COD), vacuum oven drying (VOD), infrared drying (IRD), microwave drying (MD), and freeze drying (FD)—on turmeric rhizomes slices. Key parameters analyzed included moisture loss, color, total phenolic content (TPC), curcumin content, antioxidant capacity and essential oil (EO) content and composition. FD demonstrated superior performance, achieving the brightest yellow color, the lowest browning index (17.07), the highest antioxidant capacity, TPC (78.90 mg GAE/g), curcumin content (3.83 %), and EO yield (4 %), with ar-turmerone and α-zingiberene as dominant components. While FD excelled in quality preservation, MD and IRD emerged as efficient alternatives due to their faster processing times and lower energy consumption, offering energy-efficient solutions for high-quality turmeric drying.

## Highlights

• This study evaluated six drying methods and their impact on turmeric's quality.
• Freeze drying kept the best color, phenolics, antioxidants, and curcumin levels.
• Sun drying caused the most quality loss, reducing color brightness and curcumin.
• Microwave and infrared drying saved time/energy with good quality retention.
• Essential oil yield was highest in freeze-dried turmeric, with ar-turmerone dominant.

## 1 Introduction

Turmeric (*Curcuma longa* L.) is a perennial plant with rhizomes, classified under the Zingiberaceae family. It is cultivated as an annual crop in tropical and subtropical regions, particularly in India and China (Jeevarathinam et al., 2021). This vibrant yellow-orange spice, obtained from its underground organ, the rhizome, has garnered worldwide attention not only as a culinary ingredient but also for its medicinal, antioxidant, and antimicrobial properties. Curcuminoids are the main components of turmeric's bioactive compounds, which include curcumin, demethoxycurcumin and bisdemethoxycurcumin. Studies indicate that curcumin, the most abundant component of turmeric, exhibits a wide range of biological activities, including cardioprotective, blood lipid-lowering, antibacterial, anti-HIV, anti-tumor, anticancer, antidiabetic, and anti-arthritis effects (Feng et al., 2023; Roney et al., 2025; Spanoudaki et al., 2024; Xue et al., 2023). The essential oil (EO) of *C. longa* is reported to contain ar-turmerone, curlone and turmerone as its major constituents (Monton et al., 2019). The final value of turmeric powder depends on the initial quality of the fresh rhizome and postharvest treatments applied (Jeevarathinam et al., 2021). Drying is one of the oldest and most common preservation methods in agricultural processing. It inhibits the activity of enzymes, microorganisms, and unwanted biochemical reactions, both enzymatic and non-enzymatic, by reducing the moisture level of food, resulting in a longer shelf life for fresh product and lower transportation costs (Mahayothee et al., 2020). However, several studies have shown that drying methods and conditions can affect the degradation of bioactive compounds (Zeng et al., 2023). The postharvest drying process, which is critical for ensuring the sustainability and marketability of turmeric, significantly impacts the preservation of its valuable compounds. The efficiency of each drying method varies depending on factors such as temperature, humidity, airflow, and drying duration. The drying method can affect the concentration, stability and bioavailability of curcumin and the amount of EO, influencing the quality and therapeutic potential of turmeric (Ray et al., 2022). Sun drying is the most widely used technique for drying turmeric rhizomes to produce and preserve them in powdered form. While this method benefits from utilizing solar energy, the most economical energy source, it is limited by its extended drying duration, leading to significant loss of curcumin and EO, non-uniformity of product quality, and a possibility of microbial attack due to high initial humidity (70–80 %) (Komonsing et al., 2022). Turmeric rhizomes dried in a microwave oven exhibited a significant reduction in drying time compared to those dried using a conventional hot air oven (Charoenchai et al., 2020). The study found that all drying methods reduced chemical constituents, particularly curcuminoids, with sun-drying showing the most significant reduction due to UV-induced degradation (Chumroenphat et al., 2021). Each method involves a trade-off among operational efficiency, cost and composition retention, emphasizing the need for an optimal drying approach that maximizes retention of curcumin and EOs. Although various methods of drying turmeric have been investigated, existing studies exhibit two key limitations: either the techniques employed are restricted in scope, or they fail to examine critical parameters such as color, essential oil content, and curcumin retention (Ray et al., 2022; Jeevarathinam et al., 2021; Chumroenphat et al., 2021; Monton et al., 2019; Gounder & Lingamallu, 2012). This research presents a systematic comparison of conventional (sun drying, conventional oven) and advanced (infrared, freeze-drying, vacuum, and microwave) drying methods for fresh turmeric, marking the comprehensive evaluation their simultaneous impact on color (as visual quality) and two key indicators: curcumin content (as the primary active compound) and essential oil (as a measure of aroma and volatility). By integrating data on phytochemical preservation and process performance, this work provides actionable insight for selecting context-optimal drying techniques in industrial applications.

## 2 Material and methods

### 2.1 Chemical reagents

All chemicals and reagents used were of HPLC or analytical grade. Curcumin standard with a purity higher than 98 %, n-alkanes (C8-C24), and the reagents exhibiting antioxidant activity were obtained from Sigma–Aldrich Co. (St. Louis, USA): Folin–Ciocalteu's reagent, DPPH (2,2-diphenyl-1-picrylhydrazyl), 2,4,6-tripiridyl-*s*-triazine (TPTZ), and ABTS (2,2′-azino-bis(3-ethylbenzthiazoline-6-sulphonic acid)). The solvents and reagents used in the HPLC analysis were obtained from Merck (Darmstadt, Germany).

### 2.2 Plant material

Turmeric (*Curcuma longa* L.) plants were cultivated hydroponically in the Spice Research Greenhouse at Tarbiat Modares University, Tehran, Iran. Healthy, uniformly sized, and mature fresh turmeric rhizomes were collected at physiological maturity (9 months after planting). The freshly harvested rhizomes were transported to the laboratory, where they underwent thorough washing to eliminate physical contaminants. Prior to drying experiments, the cleaned rhizomes were mechanically sliced into uniform 3-mm-thick cylindrical sections using a precision cutter.

### 2.3 Moisture content

To determine the initial moisture content, 100 g of turmeric rhizomes were dried in an oven (Binder, 7200 Tuttlingen, Germany) at 105 °C until their weight stabilized (Ray et al., 2022). During the drying experiments, the samples were weighed using an analytical balance with precision of ±0.1 mg (Sartorius, TE214S, Germany). This procedure was carried out in triplicate. The average moisture content, calculated from the three replicates, was found to be 79.4 ± 0.9 % based on fresh weight.

### 2.4 Drying methods and determination of moisture loss

Fresh  turmeric rhizomes were sliced with a knife and divided into three groups, each weighing 200 g. Moisture loss was determined by weighing the turmeric at various intervals during drying using various techniques until the moisture content dropped below 10 %. Each drying method was repeated three times for accuracy.

#### 2.4.1 Sun drying (SD)

For SD, turmeric slices were placed on a linen cloth in a steel tray exposed to sunlight at a temperature of (30–35) ± 2 °C and a relative humidity of 30 ± 5 % (Gümüşay et al., 2015).

#### 2.4.2 Conventional oven drying (COD)

A convection oven (Binder, 7200 Tuttlingen, Germany), measuring 100 × 120 × 60 cm (Width × Height × Depth) was used to dry the samples at a constant temperature of 50 °C (Ray et al., 2022).

#### 2.4.3 Vacuum oven drying (VOD)

Drying was performed in a vacuum oven following the method described by Gümüşay et al. (2015), with slight modifications. Turmeric slices were distributed on stainless steel trays and dried in a vacuum oven (Memmert, GMBH D-91126, Germany). A fixed vacuum level of 30 mbar and a temperature of 50 °C were used to dry the samples.

#### 2.4.4 Infrared drying (IRD)

For IRD, a custom-made dryer equipped with two 250 W infrared lamps inside a light and heat insulated chamber was used. Turmeric slices were arranged in a single layer on a glass tray (40 × 30 cm) and exposed to infrared radiation at an intensity of 3.7 W/m^2^. Weight measurements were recorded at 15-min intervals, and drying continued until the samples reached the target moisture content (An et al., 2016).

#### 2.4.5 Microwave drying (MD)

Microwave treatment was applied in a Samsung microwave oven (Model M945) at a power setting of 500 W. The weight of the samples was measured every 5 min (Ray et al., 2022).

#### 2.4.6 Freeze drying (FD)

Before freeze drying, turmeric rhizome slices were frozen at −80 °C for 24 h and then freeze-dried using a freeze dryer (Scanlaf, Model 9 Pro-55, Denmark) with a condenser temperature of −58 °C and a pressure of 0.1 mbar (An et al., 2016).

The dried samples were ground into powder using an electric grinder (TSM6A011W, BOSCH, Germany), sieved through a 25-mesh sieve, and then stored at −20°Ϲ prior to measurements.

### 2.5 Energy consumption

The energy consumtion of each device in each drying method was calculated based on operation time and power rating, and results are presented in Table 1:

**Table 1:** Energy consumption of dryer devices.

Devices	Device's power (W)	Duration of use (min)	Energy consumption (kW/h)
Conventional oven dryer	2000	469	15.63
Vacuum dryer	2000	355	11.83
Infrared dryer	500	90	0.75
Microwave dryer	1000	28	0.46
freeze dryer	1870	610	19.01

### 2.6 Color characteristics

The color values of fresh and dried samples were measured using a Colorimeter (CM-2600d, Konica Minolta, Japan) in CIE L* (the lightness/darkness coefficient), a* (the redness/greenness coefficient), b* (the yellowness/blueness coefficient) coordinates (Ray et al., 2022). A clean, scratch-free white cap was used to calibrate the device. Primary color values were determined by averaging measurements taken from 20 pieces of fresh turmeric slices. Three replications of dried powder were measured. Chroma (C), hue angle (H°), total color change (ΔЕ) and browning index (BI) were calculated using Eqs. (1), (2), (3), (4), respectively: (all parameters—except for hue angle (h°), which is measured in degree—are dimensionless.)

(1) $$C^{*}={\left(a^{*2}+b^{*2}\right)}^{1/2}$$

(2) $$H^{{}^{\circ}}=\tan^{-1}\left(b^{*}/a^{*}\right)$$

(3) $$\Delta E={\left(\Delta L^{*2}+\Delta a^{*2}+\Delta b^{*2}\right)}^{1/2}$$

Here, ΔE represents the difference in each parameter between fresh and dried samples.

(4) BI=[100(x−0.31(]/0.17

$$X=\left(a^{*}+1.75\;L^{*}\right)/\left(6.645\;L^{*}+a^{*}-3.01{\text{2b}}^{*}\right).$$

### 2.7 Extraction method

Sample extraction was carried out following the method outlined by Komonsing et al. (2022): 0.1 g of dried turmeric powder was mixed with 10 mL of 70 % methanol using a vortex mixer for 1 min. The mixture was then treated ultrasonically (Elmasonic S60 H, Germany) for 15 min, with the water temperature maintained below 30 °C. After ultrasonication, the mixture was centrifuged at 4600 rpm and 4 °C for 10 min using a refrigerated high-speed centrifuge (Sigma 3-30k, Germany). The residue was re-extracted twice more, each time with 10 mL of methanol, in the ultrasonic bath for 15 min. The supernatant was collected in a volumetric flask, and the extract was stored in an amber vial at −18 °C overnight before analysis.

### 2.8 Total phenolic content (TPC)

Total phenolic content (TPC) was measured using the Folin-Ciocalteu method (Komonsing et al., 2022) with slight modifications. Briefly, 200 μL of the extract was combined with 1.0 mL of a 10-fold diluted Folin-Ciocalteu reagent in a dark vial. After resting for 5 min, 1.6 mL of a 7.5 % Na₂CO₃ solution was added. The resulting mixture was stored in a dark place for 120 min. The absorbance was recorded at 765 nm using a UV–Vis spectrophotometer (G6860A, Agilent Cary 60). A calibration curve was plotted using gallic acid solutions with concentrations of 0, 5, 10, 15, 25, 50, 100, 150, 200, 250 and 500 mg L^−1^. TPC was calculated in mg gallic acid equivalent (GAE) per gram of dry matter.

### 2.9 Determination of antioxidant capacity

The antioxidant capacity was assessed using three methods for electron transfer reaction: the DPPH radical scavenging assay, the ferric reducing antioxidant potential (FRAP), and the ABTS radical scavenging capacity assay, as outlined by Komonsing et al. (2022). A calibration curve was constructed with a Trolox standard solution at concentrations of 0, 10, 20, 50, 100, 150, 200, and 250 mg/L. Results were reported as mg Trolox equivalent per gram of dry matter (mg TE/g DM).

#### 2.9.1 Ferric reducing antioxidant potential (FRAP)

In the FRAP assay, 150 μL of turmeric extract was mixed with 2850 μL of FRAP solution. Following 10 min of incubation, the absorbance was recorded at 593 nm.

#### 2.9.2 ABTS radical scavenging capacity assay (ABTS)

In the ABTS assay, a radical cation (ABTS•^+^) solution was created by combining 5 mL of 7 mM ABTS solution with 5 mL of 2.6 mM K₂S₂O₈ solution, then incubating it in the dark for 16-h. Prior to use, this ABTS•^+^ solution was diluted with 300 mL of 60 % methanol to attain an absorbance of 1.100 ± 0.020 at 734 nm. Following this, 150 μL of turmeric extract was added to 2850 μL of the ABTS•^+^ solution, and the mixture was allowed to react for 120 min in the dark. The absorbance was measured at 734 nm.

#### 2.9.3 DPPH radical scavenging assay

For the DPPH assay, 100 μL of turmeric extract was combined with 3.9 mL of 0.6 mM DPPH solution. A control was created using 100 μL of 80 % methanol. The mixture was kept in the dark for 120 min, and the absorbance was measured at 517 nm.

### 2.10 Determination of curcumin content

The determination of curcumin was performed using a high-performance liquid chromatography (HPLC) (600 Waters Dual Absorbance UV detector-USA) as described by Mahayothee et al. (2020), with some modifications. Injection was done through a 7725i Rheodyne manual injector equipped with a 20 μL loop. Chromatographic separation was carried out on a C18 column (Develosil ODS-UG-5, 4.6 mm × 250 mm, 5 μm particle diameter) from Nomura Chemical (Japan), with a runtime of 15 min. Data acquisition and integration were managed with Empower Pro software. Mobile phase A consisted of water and mobile phase B consisted of methanol. Chromatographic separations were carried out under isocratic conditions with a mobile phase of water: methanol (30,70) at a flow rate of 1 mL/min and room temperature. Detection was performed using a UV detector set at 425 nm. Curcumin content was quantified using a calibration curve (2–500 mg/L) and expressed as mg curcumin/g dry matter.

### 2.11 Essential oil isolation

Essential oil (EO) was extracted by hydro-distillation utilizing a Clevenger-type apparatus. Dried samples were ground, and 30 g of the ground material was placed in a 1000 mL round-bottom flask containing 600 mL of water. The distillation was performed by boiling the mixture for 5 h. The oil yield was presented as mL/100 g dry matter. The extracted EO was dehydrated with anhydrous Na_2_SO_4_ and stored in an amber glass bottle at 4 °C until analysis. Each distillation was performed in triplicate (Ebadi et al., 2015). The volume of volatile oil was measured, and its content was expressed as a percentage (%*V*/*W*) using Eq. (5):

(5) $$\text{Volatile oil content}\;\left(\%\right)=\left(\text{Volume of volatile oil}/\text{Initial weight of turmeric powder}\right)\times 100$$

### 2.12 Gas chromatography (GC) and gas chromatography–mass spectrometry (GC–MS)

GC analysis was performed on an Agilent Technologies 7890B system equipped with a flame ionization detector (FID), and an HP-5 capillary column (30 m × 0.32 mm i.d. × 0.25 μm film thickness). High-purity helium was used as the carrier gas at a flow rate of 1.1 mL/min, with a split ratio of 1:100. The oven temperature was programmed as follows: initial hold at 60 °C for 2 min, then ramped to 280 °C at 5 °C/min and held for 0 min. The injector and detector temperatures were maintained at 250 °C and 280 °C, respectively (Zhang, Yang, Chen, et al., 2017; Zhang, Yang, Wei, et al., 2017).

GC–MS analysis was conducted using an Agilent Technologies 7890 A system coupled with a mass spectrometer, employing an HP-5 column (30 m × 0.25 mm × 0.25 μm film thickness). The temperature program was set to 60–250 °C at 5 °C/min, with the transfer line held at 250 °C. The ionization energy was 70 eV, and helium was used as the carrier gas at 1.1 mL/min (split ratio: 1:100). The constituents of essential oils were identified by comparing their mass spectra with Wiley 7.0 and Adams libraries and were confirmed via retention indices (RIs) matched with authentic compounds. RIs were calculated using n-alkanes (C8–C24, Sigma-Aldrich) (Adams, 2007). The composition was reported as relative percentages, with 53 compounds identified.

### 2.13 Statistical analysis

Data from the completely randomized design (CRD) experiment were analyzed using SAS (version 9.4.1). Treatment effects were evaluated through one-way analysis of variance (ANOVA), with significance determined at α = 0.05. Assumptions of normality (Shapiro-Wilk test) and homogeneity of variance (Levene's test) were confirmed (*p* > 0.05 for both). Post-hoc comparisons were conducted using Fisher's least significant difference (LSD) test. Effect sizes were calculated as η^2^ (eta-squared), and results are presented as means ± standard error of the mean (SEM) with 95 % confidence intervals (CIs). Differences were considered statistically significant at *p* < 0.05.

## 3 Results and discussion

### 3.1 Drying curves and moisture loss

**Fig. 1:**
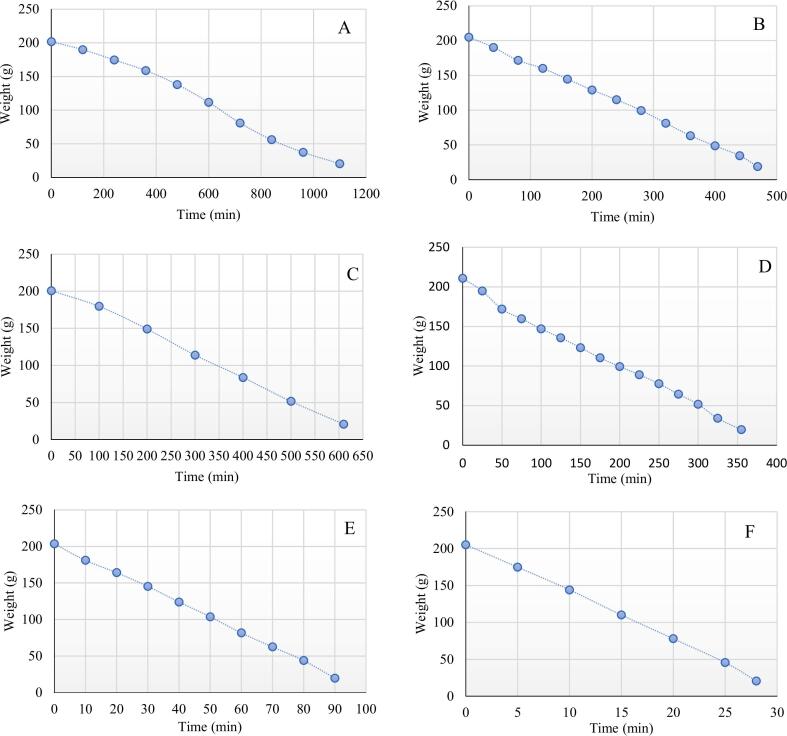
Drying rate in all methods. A: Sun drying (SD), B: Conventional oven drying (COD), C: Freeze drying (FD), D: Vacuum oven drying (VOD), E: Infrared drying (IRD), F: Microwave drying (MD).

**Fig. 2:**
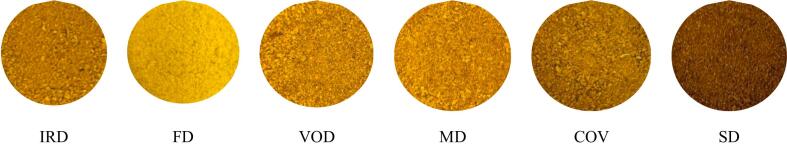
Dried turmeric samples.

The moisture loss curves of sliced turmeric (Fig. 1) reveal striking differences in drying efficiency across methods; MD emerged as the fastest technique, achieving complete drying in just 28 min—39× quicker than SD (1100 min) and 22× faster than FD (610 min). IRD ranked second (90 min), followed by VOD (355 min) and COD (469 min). Volumetric heating in microwave drying penetrates the turmeric rhizomes uniformly, rapidly evaporating moisture without excessive surface hardening (Zeng et al., 2023). MD energy efficiency with reduces processing time by ∼98 % compared to SD, minimizing thermal degradation risks (Mohamed Ahmed et al., 2024). The moisture loss curves of sliced turmeric from different drying methods are shown in Fig. 1. The quickest drying time was 28 min, achieved by MD (Fig. 1-F), followed by IRD at 90 min (Fig. 1-E), VOD at 355 min (Fig. 1-D), COD at 469 min (Fig. 1-B), FD at 610 min (Fig. 1-C), and SD at 1100 min (Fig. 1-A). SD is commonly used for drying turmeric rhizomes, enabling their preservation and subsequent processing into powdered form. However, the quality of turmeric obtained through this method is poor. Although this approach takes advantage of solar energy, the most cost- effective energy source available, it is a labor-intensive process with long drying times, insufficient sunlight, undesirable color changes, deterioration from insects and rodents, and unexpected rain, among other drawbacks (Borah et al., 2017). (See Fig. 2.)

Nowadays, microwave heating has gained popularity for drying agricultural products because of its ability to provide uniform volumetric heating, shorten drying and processing time, enhance the preservation of product quality, and improve energy efficiency (Table 1). Beyond turmeric, several studies have highlighted the favorable results of microwave drying on various fresh products such as ginger and pepper (Mohamed Ahmed et al., 2024; Zeng et al., 2023). Taskın and Izlı (2019) reported that the fastest microwave drying method for turmeric took 60 min and produced results closest to the fresh sample's color, while freeze drying required 600 min. Additionally, IRD method has many advantages over conventional drying methods (solar, oven, and vacuum) because its direct energy transfer to the product rapidly increases the drying temperature. By controlling process conditions, IRD reduces the drying time, consumes less energy (Table 1) for water evaporation and ensures uniform temperature distribution, resulting in a higher-quality final product (Jeevarathinam et al., 2022). In our study, the use of FD, which is a non-thermal method, despite being time-consuming at 610 min, resulted in very good quality, especially in color, of the dried product. Chumroenphat et al. (2021) reported that, with minimal modifications, freeze drying is more suitable for drying turmeric than hot air or sun drying.

### 3.2 Color parameters

**Fig. 3:**
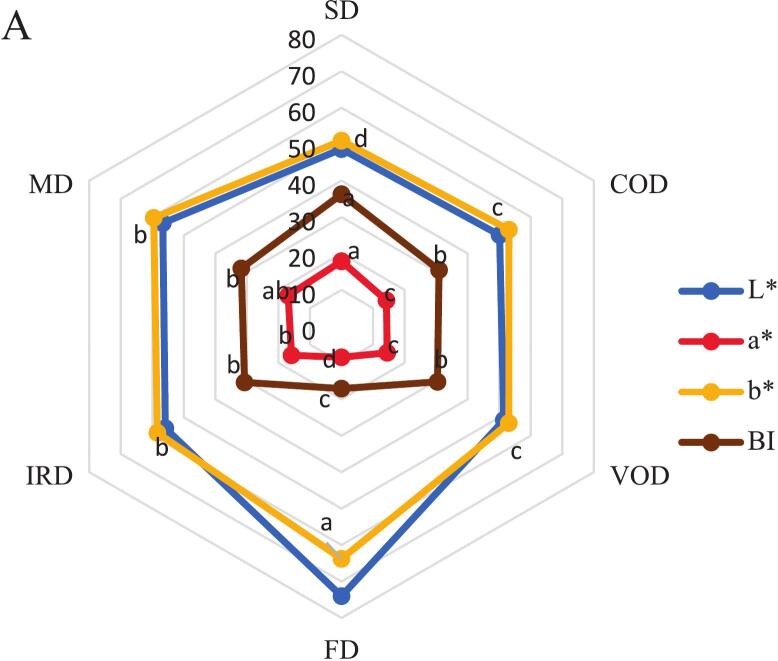

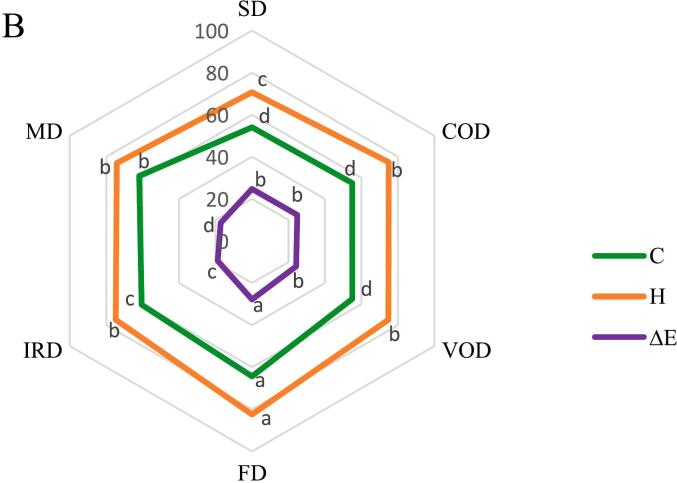
Color values of dried turmeric with various drying methods, A: (L^⁎^, a^⁎^, b^⁎^ and BI), B: (C, H°, ΔE). Different lowercase letters indicate significant difference between drying methods (*p* < 0.05).

The CIELAB color parameters (L*, a*, b*) exhibited significant variations (*p* < 0.05) across the different drying techniques. These differences are illustrated in Fig. 3-A. Freeze-dried (FD) samples exhibited optimal color retention with: highest brightness (L*) 74.0 and yellowness (b*) 63.8, lowest redness (a*) 8.5, maximal chroma (C) 64.3 indicating vibrant color, superior hue angle (H°) 82.4°, closest to ideal yellow. Jeevarathinam et al. (2021) reported findings consistent with our results, observing comparable color parameters L* (52.5) and a* (18.6) for IRD samples. While our values differed slightly L* (55.9) and a* (15.9). These systematic differences may originate from variations in raw material properties or drying protocol implementation. The parameter C indicates color intensity, where a higher value results in a more saturated and vibrant color. The hue angle (H°) is a key measure of food color, with values of 0 or 360 representing red, while angles of 90, 180, and 270 correspond to yellow, green, and blue, respectively; in essence, the hue angle signifies the dominant color. Sun-dried (SD) samples showed severe quality degradation: lowest L* (48.7), b* (51.0), C (54.0) values and H° (70.7), highest a* (17.9) and browning index (BI) 36.3. Confirming our results, Mahayothee et al. (2020) reported the dried turmeric samples showed significantly lower b* and C values in solar and sun drying compared to hot air drying, indicating greater color degradation. This variation is attributed to the reduction of curcuminoid content resulting from light degradation, ultraviolet radiation and temperature fluctuations (Fig. 3-A). In the food industry, the browning of fresh product presents a significant challenge and is a leading factor in quality degradation during postharvest handling. This browning process is primarily driven by enzymatic reactions, a mechanism that is widely understood. The main causes of color deterioration include intense browning processes such as the Maillard reaction, oxidation, and alterations in surface morphology (Ray et al., 2022). No significant difference (*p* > 0.05) in browning rates was observed among MD, COD, VOD, and IRD methods, highlighting the critical role of temperature duration rather than absolute temperature in color degradation. The color change from light yellow to brownish yellow with decreasing brightness is attributed to the alkaline decomposition of curcumin into ferulic acid and feruloyl methane (Jeevarathinam et al., 2021). In our results (Fig. 3-B), the most significant color changes (ΔE) were observed in the FD sample, attributed to a notable increase in the brightness value (L*) and a decrease in the a* value when compared to the fresh sample. The increase in brightness may be linked to the increase in porosity and uniformity of the freeze dried turmeric powder particles, resulting in consistent light reflection from a transparent yellow surface (Ray et al., 2022). Consequently, FD emerges as the most effective method for maintaining color integrity and preventing the deterioration of color properties in turmeric samples.

### 3.3 Total phenolic content (TPC)

**Fig. 4:**
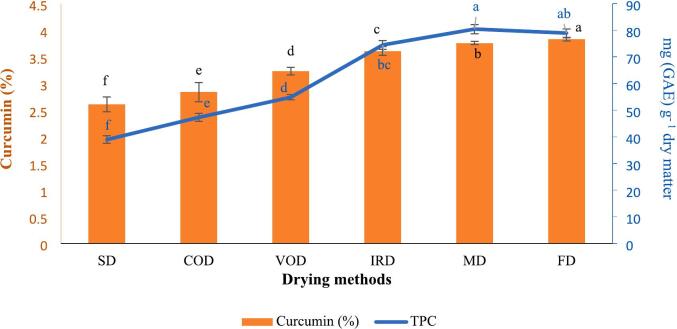
Total phenolic and Curcumin content in turmeric with various drying methods. Different lowercase letters indicate significant difference between drying methods (p < 0.05).

The TPC of turmeric dried by various methods is shown in Fig. 4. The lowest TPC was found in turmeric dried in sunlight, using 38.9 mg (GAE)/g dry matter, while the highest values were observed in turmeric dried by microwave, freeze dryer, and infrared at 80.4, 78.9 and 74.5 mg (GAE)/g dry matter, respectively. However, no significant difference (p > 0.05) was noted between MD and FD methods, nor between FD and IRD methods. Consistent with our results, An et al. (2016) observed the highest total phenolic content in ginger dried by freeze-drying (13.83 ± 0.31 mg GAE/g d.w) and infrared methods (11.35 ± 0.66 mg GAE/g d.w). Contrary to our findings of curcumin reduction, Chumroenphat et al. (2021) observed elevated total phenolics in sun-dried turmeric. Their study explained this phenomenon as resulting from photodegradation-derived phenolic acids (vanillin, ferulic acid, vanillic acid) compensating for the curcumin loss. Conversely, in MD and IRD methods, the heat generation from molecular vibrations broke covalent bonds in polyphenols, releasing small phenolic compounds and thereby increasing the total phenol content (Park et al., 2022). General, drying increases the porosity of the sample and improves dissolution during the extraction process. Comparative analysis across multiple studies consistently demonstrates freeze-drying's superior preservation of phenolic compounds. Ghafoor et al. (2020) reported maximum total phenol retention in freeze-dried ginger, while Gümüşay et al. (2015) confirmed similar advantages for both tomato and ginger samples when compared to conventional drying techniques. These observations were quantitatively supported by Zhao et al. (2021), whose statistical analysis revealed significantly higher phenolic content (*p* < 0.05) in freeze-dried persimmon slices relative to hot air-drying at all tested temperatures (60, 70, and 80 °C). The collective evidence underscores freeze-drying as the most effective method for phenolic compound preservation across diverse plant matrices.

### 3.4 Curcumin content

Our HPLC analysis revealed significant variations in curcumin content across different drying methods (Fig. 4). Freeze-dried (FD) and microwave-dried (MD) samples showed the highest curcumin retention (3.8 %), while sun-dried (SD) samples exhibited the lowest concentration (2.6 %). These findings align with previous studies demonstrating similar patterns of curcumin degradation under different drying conditions. Mahayothee et al. (2020) reported 64 % curcumin loss in greenhouse solar drying and 85 % loss in direct sunlight drying of Cassumunar ginger, consistent with our observation of maximum degradation in SD samples. Similarly, Chumroenphat et al. (2021) found SD caused the most significant reduction (72 %) among primary curcuminoids, followed by hot-air (61 %) and freeze-drying (55 %). The observed variations stem from multiple interacting factors. As a light-sensitive phenylpropanoid compound, curcumin undergoes photodegradation into phenolic derivatives including vanillin, vanillic acid, and ferulic acid when exposed to UV radiation (Mahayothee et al., 2020). Chumroenphat et al. (2021) demonstrated significantly higher concentrations of curcumin degradation products in sun-dried samples compared to freeze-dried samples, with vanillin levels approximately 5-fold greater, vanillic acid 3-fold higher, and ferulic acid 2.8-times more abundant. These marked increases in phenolic compounds provide direct chemical evidence of extensive curcumin degradation occurring during sunlight exposure. Li et al. (2016) demonstrated this vulnerability, showing 50 % degradation of pure curcumin after 24-h UV exposure. SD subjects curcumin to simultaneous light, thermal, and oxidative stress, explaining its poor performance in our study and literature reports.

Temperature-dependent enzymatic activity further influences curcumin stability. At moderate temperatures (30–45 °C), polyphenol oxidase (PPO) remains active, accelerating curcuminoid degradation while promoting vanillin formation (Chumroenphat et al., 2021). This explains why conventional oven drying (COD) and vacuum oven drying (VOD), despite protecting against light exposure, still showed suboptimal results due to prolonged processing times. Conversely, MD and infrared drying (IRD) achieved better preservation through rapid dehydration that inactivates PPO (Mahayothee et al., 2020). The superior performance of FD results from combined enzyme inhibition at sub-zero temperatures and oxidative protection under vacuum (An et al., 2016). These findings collectively demonstrate that optimal curcumin preservation requires minimizing three degradation pathways: (1) photochemical through light exclusion, (2) enzymatic via rapid drying or low temperatures, and (3) oxidative through oxygen reduction. Our results corroborate existing literature while providing specific quantification of curcumin retention under controlled drying protocols.

### 3.5 Correlation between color values and curcumin content

The correlation between color values and curcumin content in turmeric powder is shown in Table 2. According to the results, among the color values, b^⁎^ and C showed the highest positive correlation with curcumin content, (*r* = 0.919) and (*r* = 0.922), respectively (*p* < 0.0001). The highest curcumin content, along with b^⁎^ and C values was observed in FD method (Fig. 3-A and B). Among the color values, L^⁎^ shows a negative correlation with a^⁎^ (*r* = −0.827) and a positive correlation with b^⁎^ (*r* = 0.907), C (*r* = 0.861) and hue angle (*r* = 0.915). Additionally, there is a significant negative correlation (*r* = −1.0) between a^⁎^ and hue angle, indicating that a smaller a^⁎^ value corresponds to a larger hue angle, bringing it closer to yellow. Chroma, which indicates color intensity, showed a significant positive correlation (*r* = 1.0) correlation with b^⁎^ values. These correlations, illustrate the relationship between appearance color characteristics and the phytochemical quality of the product, serving as a simple method to differentiate the quality of turmeric powder from various drying methods. Therefore, it can be expected that a bright yellow color indicates higher curcumin content in turmeric powder.

**Table 2:** Pearson Correlation Coefficients, *N* = 18, Prob > |r| under H0: Rho = 0.

Variable	L*	a*	b*	C	H	Curcumin
L*	1.0	−0.827 <0.0001	0.907 <0.0001	0.861 <0.0001	0.915 <0.0001	0.757 0.0003
a*	−0.827 <0.0001	1.0	−0.614 0.0068	−0.505 0.0327	−0.965 <0.0001	−0.460 0.0578
b*	0.907 <0.0001	−0.614 0.0068	1.0	0.991 <0.0001	0.793 <0.0001	0.919 <0.0001
C	0.861 <0.0001	0.505 0.0327	0.991 <0.0001	1.0	0.705 <0.0011	0.922 <0.0001
H	0.915 <0.0001	−0.965 <0.0001	0.793 <0.0001	0.705 <0.0011	1.0	0.659 0.0029
Curcumin	0.757 0.0003	−0.456 0.0578	0.919 <0.0001	0.922 <0.0001	0.659 0.0029	1.0

### 3.6 Antioxidant capacity

**Fig. 5:**
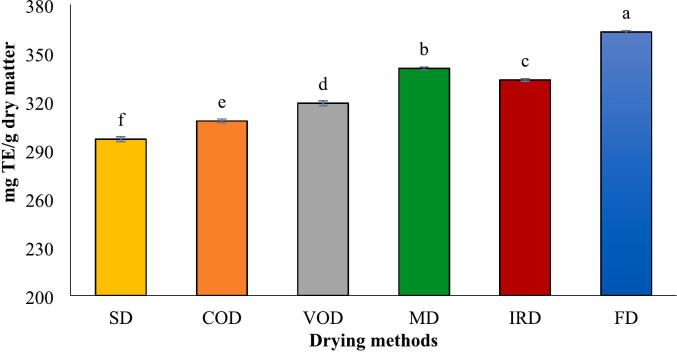

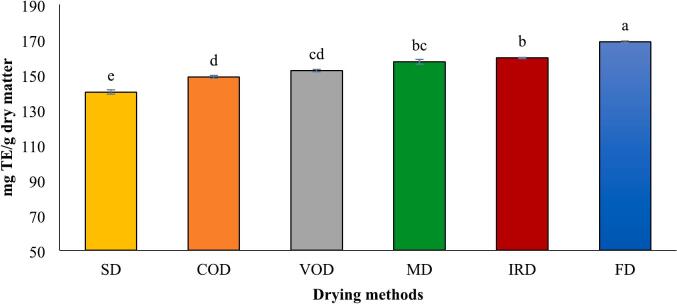

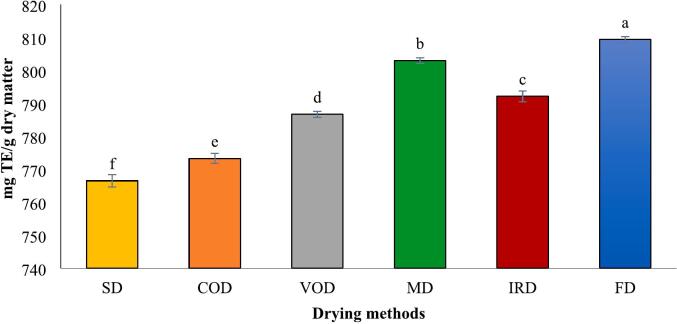
Antioxidant activities measured by means of ABTS^.+^ (A), DPPH radical scavenging (B) and FRAP (C), analyses in turmeric with different drying methods. Different lowercase letters indicate significant difference between drying methods (p < 0.05).

The antioxidant activity of dried turmeric was evaluated using three methods: ABTS, DPPH, and FRAP, with results expressed as Trolox equivalents (TE). The values for dried turmeric rhizomes ranged from 766.52 to 809.40 mg TE/g dry matter for ABTS, 140.13–168.72 mg TE/g dry matter for DPPH, and 296.70–362.88 mg TE/g dry matter for FRAP, respectively (Fig. 5 A-C). These findings illustrated that the drying method significantly influenced antioxidant activity, with FD showing the highest activity and SD the lowest. The ABTS radical scavenging assay measures the reduction of the pre-formed radical cation ABTS·^+^ upon adding turmeric extract as an antioxidant. The decolorization degree was assessed spectrophotometrically at 734 nm.

The ABTS inhibitory activity of various drying methods is depicted as Trolox-equivalent antioxidant capacity in Fig. 5-A. Across all drying techniques, the ABTS values (in TE) exceeded those obtained from the FRAP method, although the overall trend remained consistent. The highest ABTS value was noted in FD samples at 809.40 mg TE/g dry matter, followed by MD samples at 803.02 mg TE/g dry matter. A commonly used technique to evaluate antioxidant activity is the DPPH free radical scavenging assay. This method involves antioxidants interacting with the DPPH radical, causing a color transition from purple to yellow, and a corresponding reduction in absorbance at 517 nm. The results from this assay were somewhat different from those obtained with the FRAP and ABTS assay (Fig. 5-B). The DPPH assay indicated similar radical scavenging activities for MD and IRD, as well as for VOD and COD, with no significant statistical differences (*p* > 0.05).

The ferric reducing antioxidant potential (FRAP) assay evaluates a sample's capacity to convert Fe^3+^ to Fe^2+^, identifiable by the color change of the Fe^2+^-TPTZ complex. Fig. 5-C displays the FRAP values for turmeric dried by various methods. Freeze-dried (FD) samples exhibited the highest reducing power at 362.88 mg TE/g dry matter, followed by microwave-dried (MD) samples at 340.44 mg TE/g dry matter. Several studies indicate alterations in antioxidant activity after drying turmeric with different techniques. Chumroenphat et al. (2021) found that freeze-drying yielded higher FRAP and DPPH values but lower ABTS activity compared to sunlight drying. Additionally, An et al. (2016) reported that freeze-dried samples showed the highest DPPH radical scavenging activity, infrared-dried samples exhibited the highest FRAP activity, and intermittent microwave & convective drying (IM&CD) samples demonstrated the highest ABTS activity. Conversely, Komonsing et al. (2022) reported no notable differences in ABTS, DPPH, or FRAP values with varying drying temperatures for dried turmeric rhizomes. Phenolic compound and curcuminoids, are well recognized for their powerful antioxidant properties. In this study, antioxidant capacites results align with previously mentioned curcumin content and total phenol, as well as the essential oil percentage discussed later. A study demonstrated strong correlations between TPC and antioxidant assays, with DPPH (R^2^ = 0.866), FRAP (R^2^ = 0.741), and ABTS (R^2^ = 0.710) all showing significant relationships (An et al., 2016). Thus, Various drying conditions, including temperature, duration, and light exposure, may significantly influence the antioxidant capacity of turmeric by altering its bioactive compounds.

### 3.7 EOs content

**Fig. 6:**
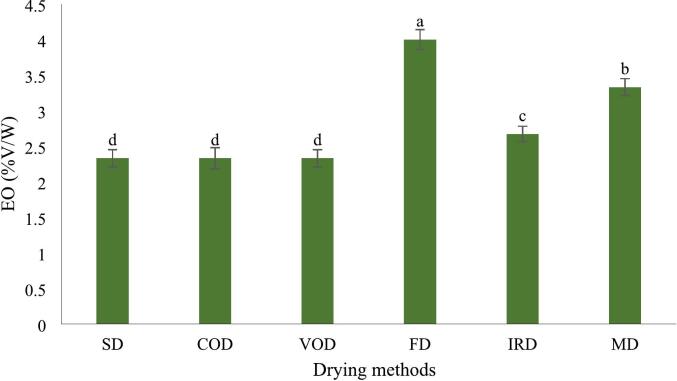
EO content in turmeric with different drying methods. Different lowercase letters indicate significant difference between drying methods (p < 0.05).

The highest EO yield of 4 % (*v*/*w*) was obtained from the FD method, followed by MD method at 3.3 % and IRD method at 2.7 % (v/w). No significant difference (p > 0.05) was observed among the drying methods of SD, COD and VOD (Fig. 6). Thus, the content of essential oil compounds was affected by drying conditions. These findings align with previous research by Ray et al. (2022), who reported the highest turmeric EO yield (1.7 % v/w) from FD, while hot-air drying yielded the lowest (0.4 % v/w). However, the present study demonstrated notably higher EO recovery, possibly due to variations in turmeric variety, drying parameters, or extraction efficiency. Interestingly, Zhang, Yang, Chen, et al. (2017) and Zhang, Yang, Wei, et al. (2017) documented even higher EO yields (4.0–5.3 %) in *C. longa* rhizomes from different Chinese regions, suggesting that geographical and genetic factors may also influence EO content. The superior EO retention in FD-treated samples can be attributed to unlike conventional drying, FD operates at low temperatures, preventing the breakdown of heat-sensitive volatile compounds (Ray et al., 2022). Lyophilization creates a highly porous matrix in turmeric rhizomes, enhancing EO release from glandular trichomes during extraction (Antal et al., 2014; Sadowska et al., 2017). Prolonged heat exposure in thermal drying methods accelerates oxidative damage, leading to EO evaporation and structural collapse of oil glands (Ray et al., 2022).

### 3.8 EOs composition

The chemical compositions of the EOs are presented in Table 3. A total of 53 components were identified through GC–MS analysis, which are terpenoids in terms of chemical structure. As indicated in Table 3, the EO of turmeric mainly consists of sesquiterpenoids (91.1–94.7 %) and a smaller proportion of monoterpenoids (4.0–6.5 %). The low boiling point and high volatility of monoterpenes led to their loss during processing steps such as drying and grinding of the rhizome (Gounder & Lingamallu, 2012). The volatile constituents of EOs were categorized into the following chemical classes: monoterpene hydrocarbons 3.8–5.7 %, oxygenated monoterpenes 0.3–1.0 %, sesquiterpene hydrocarbons 12.8–43.6 % and oxygenated sesquiterpenes 50.0–81.9 % (Table 3).

**Table 3:** The contents of chemical compositions (mg/g raw material) in essential oils from 6 drying methods.

No.					Peak Area %					
	RT^a^	Compound identified^b^	Ref. RI^c^	Cal. RI^d^	SD	COD	VOD	IRD	FD	MD
1	6.7	α-Pinene	938	932	0.1	t	0.1	t	t	t
2	7.061	Camphene	952	946	t	t	t	t	t	t
3	8.042	*β*-Myrcene	991	988	0.1	0.1	0.1	0.1	0.1	0.1
4	8.48	*α*-Phellandrene	1007	1002	2.9	2.1	2.5	2.5	1.0	2.7
5	8.585	3-Carene	1013	1008	t	t	t	t	t	t
6	8.743	*α*-Terpinene	1018	1014	t	t	t	t	0.1	t
7	8.961	*o*-Cymene	1025	1022	0.6	0.4	1.2	1.0	0.1	0.4
8	9.076	Limonene	1030	1024	0.3	0.2	0.3	0.3	0.1	0.2
9	9.152	Eucalyptol	1033	1026	0.9	0.1	0.7	0.6	0.5	0.4
10	9.859	*γ*-Terpinene	1058	1054	0.1	0.1	0.1	0.1	t	0.1
11	10.694	Terpinolene	1089	1086	1.1	0.8	1.3	1.1	2.1	1.2
12	11.319	*cis* -Thujone	1107	1101	t	t	t	t	t	–
13	12.317	Camphor	1146	1141	–	t	t	t	–	–
14	13.492	*α*-Terpineol	1192	1186	t	t	t	t	t	t
15	16.191	Thymol	1292	1289	t	t	t	t	t	t
16	17.363	*δ*-Elemene	1339	1335	t	–	–	–	t	–
17	18.252	*α*-Copaene	1372	1374	t	t	t	t	–	t
18	18.758	*β*-Elemene	1393	1389	0.1	–	–	–	0.2	t
19	19.069	Sesquithujene	1405	1405	0.2	0.1	t	t	0.2	t
20	19.336	*α-cis*-Bergamotene	1416	1411	0.1	t	–	–	0.1	–
21	19.509	*α*-Santalene	1422	1416	9.1	3.1	6.8	4.0	9.7	4.8
22	19.828	*α-trans*-Bergamotene	1436	1432	1.5	t	t	t	1.6	t
23	20.122	*epi-β*-Santalene	1448	1445	0.7	0.1	0.2	0.1	0.8	0.2
24	20.298	*(E)-β*-Farnesene	1455	1453	2.5	0.4	0.7	0.5	2.7	0.6
25	20.991	*γ*-Curcumene	1483	1481	1.6	1.6	1.9	1.6	1.7	1.4
26	21.044	Germacrene D	1485	1484	0.9	t	–	t	1.0	–
27	21.374	*α*-Zingiberene	1497	1493	10.3	3.0	2.4	2.7	11.4	3.5
28	21.62	*β*-Bisabolenol	1508	1505	3.2	0.6	0.6	0.5	3.6	0.7
29	21.768	*(Z-)-γ*-Bisabolene	1516	1514	0.4	–	t	t	0.4	t
30	21.89	*7-epi-α*-Selinene	1521	1520	0.7	–	t	–	1.1	0.1
31	22.046	Sesquiphellandrene	1526	1521	6.3	3.0	2.7	2.5	8.0	3.0
32	22.166	*(E)-iso-γ*-Bisabolene	1532	1528	0.8	0.2	0.2	0.2	0.9	0.2
33	22.233	*(Z)*-Nerolidol	1535	1531	1.0	–	0.2	–	1.2	0.1
34	23.177	Germacrene D-4-ol	1577	1574	t	0.3	0.3	–	t	0.3
35	23.26	Spathulenol	1580	1577	1.9	0.5	0.6	0.5	2.6	0.5
36	23.487	*ar*-Turmerol	1591	1582	0.6	0.4	0.4	0.3	0.6	0.3
37	23.564	*ar*-dihydro-Turmerone	1594	1595	0.8	0.2	0.2	0.19	1.2	0.2
38	23.805	Guaiol	1604	1600	0.8	1.4	1.2	1.2	1.1	1.3
39	23.909	Curzerenone	1610	1605	0.4	0.6	0.6	0.6	0.4	0.6
40	24.007	*cis*-Isolongifolanone	1614	1612	0.8	0.7	0.6	0.5	0.7	0.5
41	24.453	zingiberenol	1633	1626	0.9	0.8	1.1	0.8	0.9	0.6
42	24.558	*γ*-Eudesmol	1638	1630	0.4	1.5	1.3	1.5	0.6	1.4
43	24.776	Hinesol	1649	1640	1.0	0.5	0.4	0.4	1.0	0.5
44	25.64	*ar*-Turmerone	1673	1668	31.8	53.3	49.3	52.2	29.6	51.0
45	26.245	*β*-Turmerone (= Curlone)	1709	1699	10.0	17.6	16.4	16.9	8.4	16.6
46	26.942	(6*R*,7*R*)-Bisabolone	1749	1740	0.7	1.0	0.9	0.8	0.6	1.0
47	27.166	2,7(14)-Bisaboladien-12-ol	1761	1760	0.7	1.1	0.9	1.4	0.3	1.4
48	27.307	Aristolone	1769	1762	0.4	0.7	0.7	0.8	0.2	0.7
49	27.495	*(E)-α*-Atlantone	1777	1777	0.5	1.2	0.9	0.8	0.6	0.8
50	27.871	*β*-Eudesmol acetate	1797	1792	t	0.2	0.1	0.3	t	0.2
51	28.237	14-hydroxy-*δ*-Cadinene	1815	1803	0.2	0.7	0.6	0.9	0.1	0.6
52	30.297	11-Acetoxyeudesman-4α-ol	1944	1938	t	t	t	t	t	t
53	33.41	9,12-Octadecadienoic acid, methyl ester	2092		0.1	0.1	0.1	0.1	t	t
Total identified					**97.5**	**98.8**	**98.7**	**98.2**	**97.6**	**98.8**
Total monoterpenoids					**6.4**	**4.1**	**6.5**	**5.8**	**4.0**	**5.5**
Monoterpene hydrocarbons					**5.4**	**3.8**	**5.7**	**5.1**	**3.4**	**5.0**
Oxygenated monoterpenes					**1.0**	**0.3**	**0.8**	**0.7**	**0.61**	**0.5**
Total sesquiterpenoids					**91.1**	**94.7**	**92.2**	**92.4**	**93.6**	**93.2**
Sesquiterpene hydrocarbons					**38.5**	**12.8**	**16.1**	**13.1**	**43.6**	**15.3**
Oxygenated sesquiterpenes					**52.6**	**81.9**	**76.1**	**79.3**	**50.0**	**77.9**

a Retention time.

b Compound listed in the order of elution from a HP-5 column.

c Retention indices (RIs) relative to n-alkanes (C8–C24) on the same column.

d Calculate Retention indices. t: trace <0.1 %.

The EOs extracted from rhizome, regardless of the drying technique used, primarily composed of oxygenated sesquiterpenes as the dominant chemical group.

**Fig. 7:**
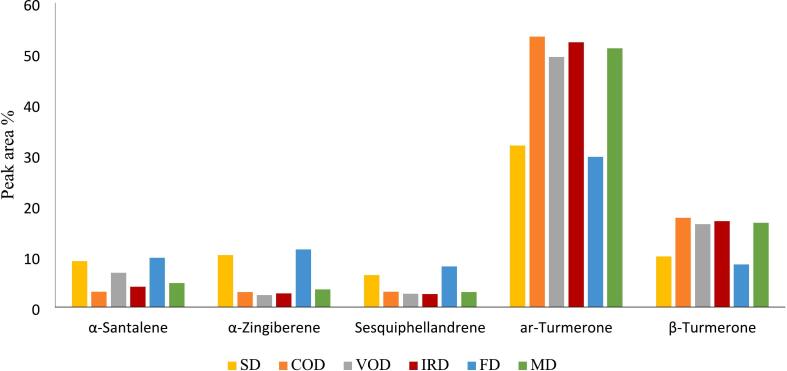
Comparative peak area (%) of α-Santalene, α-Zingiberene, Sesquiphellandrene, ar-Turmerone and β-Turmerone in different EOs by GC-FID.

**Fig. 8:**
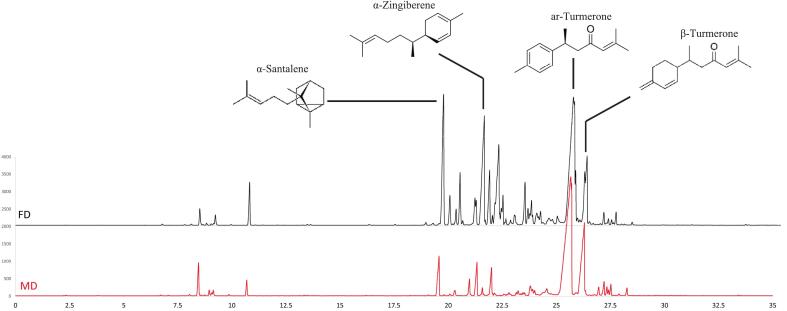
Chromatogram obtained from GC-FID screening of the EOs of dried *C. longa* rhizomes via FD and MD methods.

However, the order of the most important components was affected by the drying method (Fig. 7). In SD method, ar-turmerone (31.8 %), α-zingiberene (10.3 %), β-turmerone (10.0 %), α-santalene (9.1 %), sesquiphellandrene (6.3 %), in COD method, ar-turmerone (53.3 %), β-turmerone (17.6 %), α-santalene (3.1 %), sesquiphellandrene (3.0 %), α-zingiberene (3.0 %), in VOD method, ar-turmerone (49.3 %), β-turmerone (16.4 %), α-santalene (6.8 %), sesquiphellandrene (2.6 %), α-zingiberene (2.4 %), in IRD method, ar-turmerone (52.2 %), β-turmerone (16.9 %), α-santalene (4.0 %), α-zingiberene (2.7 %), sesquiphellandrene (2.5 %), in FD method, ar-turmerone (29.6 %), α-zingiberene (11.4 %), α-santalene (9.7 %), β-turmerone (8.4 %), sesquiphellandrene (8.0 %), and in MD method, ar-turmerone (51.0 %), β-turmerone (16.6 %), α-santalene (4.8 %), α-zingiberene (3.5 %), sesquiphellandrene (3.0 %), respectively, had the highest content among other components of the extract (Fig. 7, Fig. 8).

Turmerone is a compound that degrades rapidly at room temperature, transforming into its more stable dimer form, ar-turmerone. Chemically, turmerone (C15H22O) appears as a light yellow substance with a UV detection wavelength of 234 nm, while ar-turmerone (C15H20O) is colorless and detectable at 239 nm. The melting points of turmerone and ar-turmerone are reported to range between 110 and 120 °C and 123–126 °C at 10 mmHg, respectively, while their boiling points being approximately 109 °C and 160 °C (Jacob & Toloue, 2013). The lowest amounts of turmerones (ar and β) were obtained from samples dried by FD and SD methods, and the highest amounts were obtained from COD, IRD, and MD methods. The FD method is performed at very low temperatures, and the drying process is slow; thus, some volatile compounds may be removed from the cell structure and lost during freezing or sublimation. SD is also done at ambient temperature, but the drying time is prolonged, and which may be cause oxidation or hydrolysis of some compounds due to exposure to UV ray, oxygen and moisture.

On the other hand, thermal methods, where the temperature is controlled and usually above ambient, have shorter drying times. Rapid and controlled heating may result in better preservation of volatile compounds because there is less time for oxidation or decomposition. In IRD and MD methods, which are rapid, volatile compounds experience less exposed to decomposition from light and oxygen. Higher than ambient temperatures in the oven proved more effective in preserving turmerones compared to relatively high temperatures in microwave and infrared. Another distinction between the low-temperature drying method, i.e., FD and SD, and other drying techniques was the significant increase in some EO components, including ar-dihydro-turmerone, spathulenol, nerolidol, β-farnesene, α-trans-bergamotene and 7-epi-α-selinene. For instance, this increase in β-farnesene was 4–6 times greater than in thermal methods (COD, VOD, IRD and MD). Drying temperature affects the yield and composition of essential oils, which are rich in terpenoids (Tibaldi et al., 2022). Due to the low temperature in the FD method, compounds sensitive to thermal degradation such as nerolidol, spatholenol are not decomposed, and even light compounds with low boiling points, such as β-farnesene and α-trans-bergamotene, are preserved. On the other hand, due to the lack of oxygen and low temperature, the structure of compounds such as ar-dihydro-turmerone and 7-epi-α-selinene does not change due to oxygenation and isomerization. Ray et al. (2022) reported that the major constituents identified were ar-turmerone (28.0–38.8 %), α-turmerone (17.6–27.0 %), β-turmerone (16.5–19.0 %), and α-phellandrene (2.5–5.2 %), with significant changes in the volatile constituents of the EO associated with the drying methods used.

Based on GC–MS analysis, the primary components identified in turmeric volatile oil were ar-turmerone 43–49 %, curlone 17–18 % and turmerone 13–16 %, respectively (Monton et al., 2019). In another study, it was found that ar-turmerone (32 %), α-turmerone (16 %), and β-turmerone (13 %) were dominant volatile compounds (Thongphasuk & Thongphasuk, 2013). Additionally, research on turmeric oil from Iran revealed that ar-turmerone 68.9 %, α-turmerone 20.9 %, and α-phellandrene 2.2 % were the most abundant volatile constituents (Asghari et al., 2010). Furthermore, another study identified 43.0 % of the EO compounds of *C. longa*, with the main components being ar-turmerone (11.8 %), zingiberene (8.6 %), β-sesquiphellandrene (6.7 %), and β-turmerone (4.1 %) (Zhang, Yang, Chen, et al., 2017; Zhang, Yang, Wei, et al., 2017). A study of 20 EOs of rhizomes obtained from different regions of China showed that 81 components were identified, with major compounds were ar-turmerone (0.9–42.8 %), β-turmerone (5.1–42.5 %), α-zingiberene (0.2–25.0 %), ar-curcumene (1.2–15.7 %) and β-sesquiphellandrene (0.05–14.89 %) (Zhang, Yang, Chen, et al., 2017; Zhang, Yang, Wei, et al., 2017). Turmeric essential oil has proven physiological and therapeutic effects due to its unique compounds. Reports indicated that some major constituents of turmeric essential oil, especially ar-turmerone, α-turmerone, zingiberene, germacrone, β-elemene, α-curcumene and β-sesquiphellandrene, having anticancer and anti-inflammatory effects (Zhang et al., 2020), hypoglycemic action, cardiovascular and antioxidant activities (Fan & Watanabe, 2022; Panigrahy et al., 2021), along with dermatological applications, neuroprotective effects, hepatoprotective properties, and antiviral and antifungal activities (Gnat et al., 2019; He et al., 2019; Ti et al., 2021).

## 4 Conclusions

This study evaluated the impact of various drying methods on the drying characteristics, color parameters, total phenolic content, curcumin retention, antioxidant capacity and EO composition of turmeric. The results demonstrated that drying methods significantly influenced turmeric quality. Sun drying (SD), while cost-effective, resulted in the poorest quality in terms of color, curcumin retention, antioxidant capacity and EO content. In contrast, microwave drying (MD) and freeze drying (FD) exhibited superior performance in preserving bioactive compounds, with MD providing the fastest drying time and FD maintaining the best color. Infrared drying (IRD) also emerged as an effective method, balancing drying efficiency and quality preservation. The strong correlation between color parameters and curcumin content suggests that color measurements can serve as a simple indicator of turmeric quality. Moreover, the strong correlation between color degradation and curcumin loss suggests that visual quality could serve as a rapid indicator of bioactive retention in industrial settings. These findings provide valuable insights for optimizing industrial turmeric drying processes to maximize quality while maintaining efficiency. Future research should further explore the influence of drying parameters on turmeric's functional properties to enhance its industrial application.

## CRediT authorship contribution statement

**Aghdas Shahimoridi:** Writing – original draft, Visualization, Software, Methodology, Investigation, Formal analysis, Data curation. **Mohammad-Taghi Ebadi:** Writing – review & editing, Supervision, Project administration, Investigation, Funding acquisition, Conceptualization. **Mahdi Ayyari:** Writing – review & editing, Formal analysis. **Yadollah Yamini:** Writing – review & editing, Formal analysis.

## Declaration of competing interest

The authors declare that they have no known competing financial interests or personal relationships that could have appeared to influence the work reported in this paper.

## Data Availability

Data will be made available on request.
